# Fibrosis severity scoring on Sirius red histology with multiple-instance deep learning

**DOI:** 10.1017/S2633903X23000144

**Published:** 2023-07-18

**Authors:** Sneha N. Naik, Roberta Forlano, Pinelopi Manousou, Robert Goldin, Elsa D. Angelini

**Affiliations:** 1 ITMAT Data Science Group, NIHR Imperial BRC, Imperial College, London, United Kingdom; 2 Heffner Biomedical Imaging Lab, Department of Biomedical Engineering, Columbia University, New York, NY, USA; 3 Faculty of Medicine, Department of Metabolism, Digestion and Reproduction, Imperial College, London, United Kingdom; 4 Department of Hepatology, Imperial College Healthcare NHS Trust, London, United Kingdom; 5 Section for Pathology, Imperial College, London, United Kingdom; 6 Telecom Paris, Institut Polytechnique de Paris, LTCI, Palaiseau, France

**Keywords:** deep-learning, histopathology, liver fibrosis

## Abstract

Non-alcoholic fatty liver disease (NAFLD) is now the leading cause of chronic liver disease, affecting approximately 30% of people worldwide. Histopathology reading of fibrosis patterns is crucial to diagnosing NAFLD. In particular, separating mild from severe stages corresponds to a critical transition as it correlates with clinical outcomes. Deep Learning for digitized histopathology whole-slide images (WSIs) can reduce high inter- and intra-rater variability. We demonstrate a novel solution to score fibrosis severity on a retrospective cohort of 152 Sirius-Red WSIs, with fibrosis stage annotated at slide level by an expert pathologist. We exploit multiple instance learning and multiple-inferences to address the sparsity of pathological signs. We achieved an accuracy of 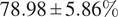 , an F1 score of 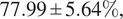 and an AUC of 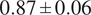 . These results set new state-of-the-art benchmarks for this application.

## Impact Statement

We demonstrate, for the first time, the capabilities of an end-to-end weakly supervised multiple-instance DL pipeline to accurately classify liver fibrosis stages between mild (stage 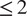 ) and severe (stage 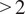 ) on whole-slide images. Such a framework works towards reducing expert annotation workload and human-induced uncertainty.

## 1. Introduction

### 1.1. NAFLD and fibrosis scoring

Non-alcoholic fatty liver disease (NAFLD) is currently the leading cause of chronic liver disease worldwide and is typically associated with obesity, insulin resistance, and diabetes^(^^1^ ^)^. In a study of over 8 million people worldwide, it was estimated that the global prevalence of NAFLD is 30% with the highest prevalence in the Middle East and South America^(^^2^ ^)^. Furthermore, in the USA, NAFLD became the first indication for liver transplantation in 2022^(^^3^ ^)^. Given the increasing worldwide incidence of obesity, it is likely that NAFLD in the global population will continue to rise in decades to come. Fibrosis stage is the main predictor of liver- and non-liver-related outcomes and fibrosis progression or non-worsening of fibrosis is the end-point in all clinical trials. Therefore, the evaluation of fibrosis progression in clinical trials is of key interest.

### 1.2. Histopathology of the liver

Liver biopsy is the reference “gold” standard for the diagnosis and staging of NAFLD^(^^4^ ^)^. The liver consists of the portal tracts (containing bile ducts and vascular branches), terminal hepatic venule, hepatocytes, and sinusoids. Histopathologists most commonly use the semi-quantitative Non-alcoholic Steatohepatitis Clinical Research Network (NASH CRN) scoring system to stage the severity of liver disease seen on biopsies^(^^5^ ^)^. The scoring system (described in detail in^(^^6^ ^,^^7^ ^)^) relies on visual signs of four pathological processes^(^^4^ ^)^: Steatosis, Lobular inflammation, Ballooning, and Fibrosis. The presence of the first three components (Steatosis, Ballooning, Lobular inflammation) are combined into an unweighted sum to generate the NAFLD Activity Score (NAS score) which ranges from 0 to 8 and is an indicator of active injury in the liver. Fibrosis is defined as the accumulation of extracellular matrix proteins (e.g., collagen) in the liver^(^^8^ ^)^. It is scored separately from the other features as it is generally considered a result of disease activity rather than a feature of active injury and is less reversible. Fibrosis is graded from 0 to 4 based on the following architectural patterns visible: Stage 0: No visible fibrosis; Stage 1a: Mild perisinusoidal fibrosis; Stage 1b: Moderate-to-severe perisinusoidal fibrosis; Stage 1c: Portal / periportal fibrosis; Stage 2: perisinusoidal and portal / periportal fibrosis; Stage 3: Bridging fibrosis between adjacent portal tracts or between a portal tract and an adjacent hepatic venule^(^^4^ ^)^; Stage 4: Cirrhosis, which can lead to cell necrosis and liver failure.

Traditional glass microscopy slides are scanned using a digital slide scanner to generate whole-slide images (WSIs). Advances in slide scanning technology and reduction in the cost of digital storage have accelerated the design of solutions for automated segmentation of disease patterns on WSIs^(^^9^ ^)^, and most recently the scoring of those patterns.

### 1.3. Deep learning in histopathology

Deep Learning (DL) classifiers are raising significant breakthroughs in WSI segmentation, scoring, and classification^(^^9^ ^–^^11^ ^)^. The vast majority of published work focuses on H&E stains for cancer datasets.

WSIs are very large images, typically of size 0.5–1 Gb per WSI. This prevents opening WSIs at full resolution on standard computers, but also loading the whole field of view at full resolution on standard GPUs to train DL architectures. Options commonly used in DL are either working with pixel-level annotations–which is time-consuming to prepare or using multiple instance learning (MIL) with annotations at the WSI level. In a MIL framework, small “tiles” are extracted from WSIs to form “bags” with a single label assigned to all bags from the same WSI and which corresponds to the WSI label (commonly called weak labeling). Features are extracted from each tile (typically using a CNN), and tile embeddings or per-tile predictions are aggregated to generate an overall prediction from the bag^(^^12^ ^)^.

MIL frameworks rely on specific strategies for tile selection, feature extraction, and multiple inference aggregation. Common MIL paradigms rely on one training “bag” per image after careful tile selection (e.g., tissue content above a threshold)^(^^12^ ^–^^14^ ^)^; data augmentations^(^^12^ ^–^^17^ ^)^; use of transfer learning from ImageNet via GoogLeNet, InceptionNet, ResNet, and MobileNet^(^^18^ ^,^^19^ ^)^ for cancer, and AlexNet and ResNet for NAFLD^(^^20^ ^,^^21^ ^)^; aggregation of tile-level inferences via max-pooling, or of tile-level features via average-pooling, attention-based or RNN based frameworks ahead of WSI-level prediction^(^^12^ ^,^^13^ ^,^^16^ ^,^^21^ ^,^^22^ ^)^.

Stain normalization is often used as a pre-processing step. Classic approaches include template color transfer, such as Reinhard stain normalization^(^^23^ ^)^, and stain-deconvolution^(^^24^ ^)^. More recently, DL architectures were proposed such as sparse auto-encoders^(^^25^ ^)^, GANs^(^^26^ ^)^, and the recent Stain Standardization Capsule (SSC) architecture^(^^27^ ^)^ which outperformed both color augmentation and state-of-the-art normalization methods on three tumor classification tasks on H&E stained datasets.

### 1.4. Specific challenges

A specific challenge faced by computational methods when scoring liver fibrosis on those Sirius-Red stained WSIs is the presence of natural collagen around the portal tracts and hepatic veins. This causes direct measures of the percentage area of collagen to be similar for different fibrosis stages^(^^28^ ^)^, and suggests that both locating and quantifying the abnormal fibrosis patterns is crucial.

Histological scoring systems used to manually assess liver biopsy, such as the NASH CRN, use a semi-quantitative approach, based on describing the localization and visual patterns of liver fibrosis, but without measuring the amount of fibrosis. Such a scoring system is very subjective and is subject to significant inter and intra-observer variability. Inter-reader weighted kappa for fibrosis staging in NAFLD has been reported approximately 0.484 from scores of three independent hepatopathologists on 687 H&E-stained biopsies of NASH patients. Intra-reader kappas in the same study were moderately higher at 0.679^(^^29^ ^)^. This is considered as modest to poor agreement (on a score of −1 for total disagreement to 1 for perfect agreement). Reported kappas vary across datasets: some show fair inter-rater agreement at 0.72–0.79 for H&E and Trichrome datasets with sizes ranging from 40 to 300+ WSIs^(^^30^ ^,^^31^ ^)^. Others report cases of a two-stage difference in fibrosis severity assigned by two independent pathologists^(^^32^ ^)^.

Such a scoring system is also insufficient, indeed, the absence of a quantitative assessment has important consequences in clinical practice as the extent of liver fibrosis is commonly associated with poor clinical outcomes in these patients. Overall, the lack of an objective and quantitative assessment of histology has an enormous impact on the interpretation of clinical trials, as it might not be sensitive enough to detect small changes in fibrosis pattern. Therefore, in order to better diagnose and monitor liver fibrosis status in clinical trials on NAFLD, there is a strong need for fast and reproducible scoring of WSIs^(^^33^ ^)^.

In this paper, we propose a MIL framework to train a weakly supervised binary fibrosis classifier to distinguish Stages 0–2 from Stages 3–4, considered as a clinically-crucial fibrosis severity transition.

## 2. Methods

Our proposed method was designed to tackle a binary classification task with the following challenges at hand: small image cohort, only image-level annotations, sparse pathological signs in large tissue samples, significant stain color variability between samples and presence of artifacts inherent to non-curated retrospective clinical data.

### 2.1. The LiFib cohort

We exploit a retrospective clinical cohort, referred to in this paper as the LiFib cohort, of 152 digitized WSIs from liver biopsy samples performed as part of routine clinical care at St Mary’s Hospital. Liver tissue was collected via needle biopsy using a percutaneous technique under ultrasound guidance. WSIs were recorded with a Hamamatsu NanoZoomer2.0 HT digital slide scanner at 40x magnification using Sirius-Red stain. Each WSI contains two to three cores of tissue. The WSI images in our cohort have a pixel size of 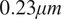 at max zoom factor of 40x. The WSIs were annotated at a slide level by an expert pathologist with 35 years of clinical experience. The four-stage fibrosis scale mentioned in the introduction is used, with the following distribution in our cohort: stage 0 = 12%, stage 1 = 23%, stage 2 = 15%, stage 3 = 35%, and stage 4 = 15%. For the binary classification task of separating mild (stage 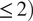 from severe (stage 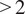 ) fibrosis, we have an equal number (n = 76) of cases per class.

Our WSIs are affected by common slide-preparation artifacts: air bubbles, knife-slicing irregularity, tissue folds, cracks, crumbling tissue (particularly for severe fibrosis cases), and fixation problems. WSIs also contain red artifacts where a vein was cut with the tissue during extraction (Figure 3a). From visual inspection, blur digitization artifacts affect only a small portion of WSIs. The LiFib dataset was not curated to exclude cases with artifacts, as those are common in routine clinical data.

We manually characterized WSI visual appearance into three categories (Figure 2): (a) green tissue on a grey background (*n* = 75), (b) green tissue on a blue background (*n* = 56), (c) yellow tissue on a grey background (*n* = 21). In all cases, collagen and fibrosis components appear as shades of red. However, in older WSIs, the stain is more faded.

We constructed five independent test sets for five-fold cross-validation, stratifying on fibrosis scores and color categories. For each fold a 70%–10%–20% train-validation-test split was used.

### 2.2. Bag construction and deep-learning pipelines

**Figure 1.**
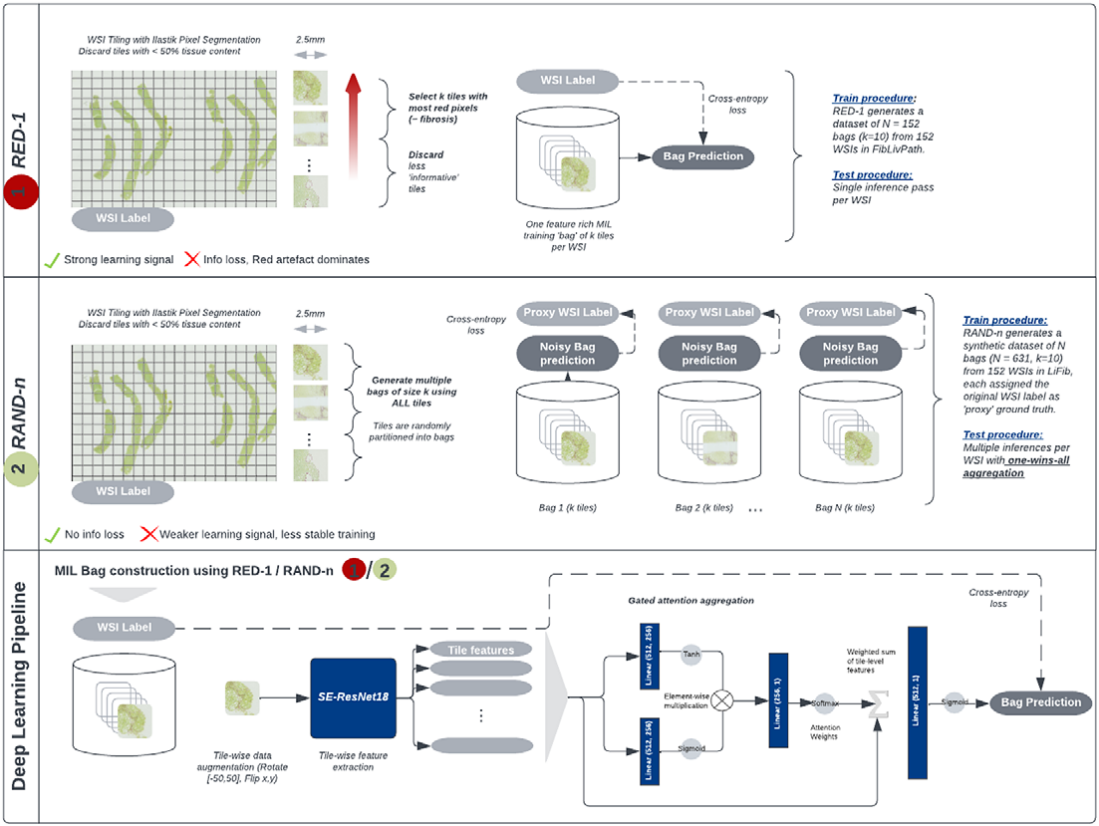
Overview of our bag construction and deep-learning pipelines for fibrosis scoring on Sirius Red histopathology WSIs. Two MIL bag construction methods are compared: (1) RED-1, which uses a priori knowledge on red appearance of fibrosis to generate 1 bag per WSI, and (2) RAND-n, which generates 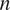 bags per WSI only based on tissue content. Deep-learning pipeline (best model): SE-ResNet18 initialized with ImageNet pre-trained weights with gated attention aggregation to output a binary label on a given bag.

An overview of the proposed bag construction and DL pipelines is provided in Figure 1.

#### 2.2.1. Tissue segmentation and tile extraction

Tissue segmentation was performed using Ilastik’s Pixel Classification workflow^(^^34^ ^)^. Background from foreground separation was trained with manual brush strokes on one WSI per color category, which led to satisfactory segmentation quality on all cases. Tiles were generated on a grid using 2.5 mm square patches at 5× magnification (pixel size 1651 × 1651), with 50% overlap. We excluded tiles from the background using a threshold on tissue content. This threshold was set to 50% except in few cases impacted by tissue crumbling for which it was iteratively decreased to retain a minimum of 10 tiles per WSI.

#### 2.2.2. Bag composition

Bags are composed of a pre-fixed number of 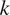 tiles and are given the original WSI label as a proxy ground-truth label. The majority of existing literature constructs a single bag per WSI that includes from thousands to tens of thousands of tiles with deemed-sufficient tissue content^(^^12^ ^–^^16^ ^)^. Unlike most cancer focused datasets, the pathological signs in NAFLD are sparse. In the LiFib cohort, for cases of mild-medium fibrosis, a large proportion of tiles generated are uninformative (containing only artifact or an absence of collagen). Consequently, using too many tiles per bag might result in a prohibitively weak learning signal in each bag. We therefore tested two alternative approaches: (1) Dataloader RED-1: we generate 1 bag per WSI using the 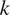 tiles with the highest percentage of red pixel content (proxy for fibrosis tissue) measured before stain normalization. (2) Dataloader RAND-n: we generate 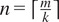 bags per WSI with 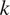 tiles randomly sampled without replacement from a pool of 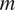 available valid tile candidates. This leads to a variable number of bags per WSI. Within each bag, tiles are downsampled to 224 × 224 pixels using bicubic interpolations and data augmentation was performed using random rotations in range 
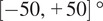
 , horizontal and vertical flips.

#### 2.2.3. MIL architectures and multiple inference aggregation

Our baseline architecture uses a ResNet18 pre-trained on ImageNet. Our advanced architecture uses the Squeeze-and-Excite^(^^35^ ^)^ module in a pre-trained SE-ResNet18 encoder to enhance robustness of features to artefacts and upweight the importance of pathological signs.

For the aggregation of tile-level features in the MIL inference, we tested max-pooling and a gated attention framework^(^^12^ ^)^, shown to outperform max-pooling of tile-level predictions on small datasets^(^^16^ ^)^.

The max-pooling architecture applies a single linear layer to the encoder outputs 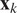 , to generate per-tile predictions 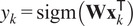 and assigns to the WSI a label equal to the maximum of per-tile predictions, 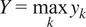 . Gated Attention aggregation (Figure 1, Equation 1) first computes attention weights per-tile 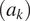 using two linear layers 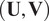 . A single WSI-level feature vector 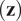 is generated from the weighted sum of tile-level features. Finally, a single linear layer 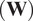 returns the final overall WSI label.
(1)

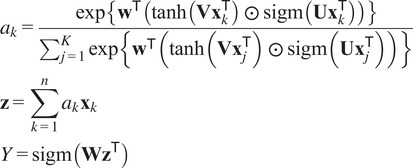



Model training and evaluation procedures are different for our two bag construction methods. (1) Dataloader RED- 1:Using 1 bag per WSI, assigned with the WSI label as proxy ground truth. During training and testing, one inference pass is run to generate the bag-level prediction. (2) Dataloader RAND-n: Using *n* bags per WSI, all assigned with the WSI label as proxy ground truths. We conduct one inference pass per bag to generate n bag-level predictions. We train using the binary cross-entropy loss between each bag prediction, and the proxy ground truth label. During training and testing, one inference pass is run per bag to generate n bag-level predictions. The final WSI prediction is set to the most severe predicted fibrosis stage across the bag-level predictions (one-wins-all aggregation).

## 3. Results

In our cohort, we extracted on average 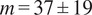 valid tiles per WSI, with 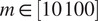 (12 WSIs with only 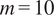 tiles). We tested bag sizes 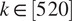 for Dataloader RED-1 and we worked with 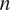 = 1 to 10 bags for Dataloader RAND-n for bag size 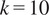 .

We report mean and standard deviation values over the 5 folds for Accuracy and F1 scores, to assess models performance and reproducibility across training sets. We also report Recall, Specificity, Precision, F2, and Matthews Correlation Coefficient (MCC) values measured on the test confusion matrix aggregated over the 5 folds.

### 3.1. Single bag approach: MIL aggregation and bag size effect

We first report results in Table 1 using Dataloader RED-1 (cf. Section 2.2) considered as the most favorable training setup guided with *a priori* knowledge on disease sign characteristics. The three most important hyperparameters to reach training stability across folds were the encoder learning rate (= 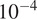 ), batch size (=6), and early stopping on the validation set. Other optimally tuned hyperparameters include weight decay (= 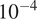 ) and Adam optimizer.

**Table 1.** Single-bag MIL architectures performance–pre-trained with ImageNet and using Dataloader RED-1 with bag size 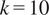 .

Architectures	Accuracy (%)	F1 (%)
ResNet18 (Maxpool)	69.03 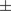 6.37	63.66 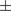 10.57
SE-ResNet18 (Maxpool)	72.37 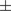 4.51	69.18 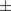 7.84
SE-ResNet18 (Gated Attention)	**74.32** 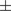 **5.38**	**70.18** 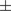 **10.00**

*Note.* Two MIL aggregation strategies are compared: max-pooling on tile inference or gated attention on tile features. Best values are indicated in bold.

From Table 1, the SE-ResNet18 with gated attention shows the best performance. We tested bag size effects on this architecture in Table 2 with 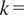 5 to 20.

**Table 2.** Effect of bag sizes 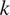 using SE-ResNet18 (Gated Attention) pre-trained with ImageNet on Dataloader RED-1 with 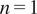 bag per WSI.

Bag size 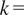	Accuracy (%)	F1 (%)	Recall (%)	Specificity (%)	Precision (%)	F2 (%)	MCC (%)
5	66.43 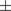 4.99	61.94 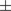 9.5	56.58	76.32	70.49	58.90	33.55
10	**74.32** 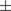 **5.38**	**70.18** 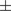 **10**	**64.47**	84.21	**80.33**	**67.12**	**49.66**
15	64.45 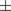 9	49.43 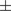 23.3	40.79	**88.16**	77.50	45.06	32.87
20	65.76 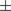 6.2	55.75 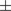 17.93	50.00	81.58	73.08	53.37	33.28

*Note.* Other hyperparameters as in Table 1. Best values are indicated in bold.

We can see in Table 2 that bag size can decrease Recall by 20% corresponding to an increase in false negatives. A bag size 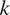 = 10 leads to the best metrics except for Specificity (−4% compared to 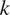 = 15) and was therefore selected for subsequent experiments. These results confirm the tradeoff that exists in MIL between bags too small that can lead to missed information (e.g., here in cases with lots of red artifacts, where thresholding on red pixels did not retain fibrosis signs of interest) and bags too large that can degrade learning capacity.

At this stage, our model suffers from two limitations likely due to some information loss when using Dataloader RED-1: (a) low Recall (64.47%) and (b) large performance variability across folds (10% standard deviation in F1). The subsequent refinement aims to improve these results.

### 3.2. Gain from multiple inference training

We compare in Table 3 our baseline that uses Dataloader RED-1 with the use of multiple bags with Dataloader RAND-n during testing only or also during training. We use the optimal bag size for Dataloader RED-1 (*k* = 10). The number of bags 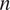 varies across WSIs, depending on the number of valid tiles with an average of 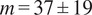 tiles extracted per WSI and a range of valid tiles per WSI from 10 to 100, hence 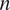 ranging from 1 to 10 for 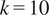 . Class balance was maintained when using RAND-n, working with a total of 313 bags for the mild fibrosis class and 318 bags for the severe fibrosis class.

**Table 3.** Comparing Dataloader RED-1 ( 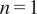 ) versus Dataloader RAND-n with bag size 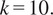

Dataloader train/test	Accuracy (%)	F1 (%)	Recall (%)	Specificity (%)	Precision (%)	F2 (%)	MCC (%)
RED-1/RED-1	74.32 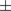 5.38	70.18 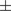 10	64.47	**84.21**	80.33	67.12	49.66
RED-1/RAND-n	73 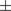 8	72 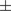 9	69.74	76.32	74.65	70.67	46.15
RAND-n/RAND-n	**78.92** 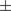 **5.8**	**77.99** 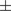 **5.64**	**75.00**	82.89	**81.43**	**76.20**	**58.08**

*Note.* RAND-n uses multiple inferences with “one wins all” aggregation. All other details as in Table 2. Best values are indicated in bold.

Using Dataloader RAND-n only at testing has a detrimental effect on Specificity and Precision (−7.9% and − 5.7%) which corresponds to an increase in false positives. On the other hand, using Dataloader RAND-n at both training and testing leads to the best performing model (except for −1.3% in Specificity), with clear gains in Accuracy of ( 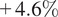 ), and F1 score ( 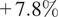 ) along with stable or decreased standard deviations across folds. It also returns an increase of +9.5% in Recall, corresponding to fewer false negatives, which could be attributed to the prevention of information missed at inference by utilizing more tiles in a WSI. This model achieves an AUC of 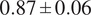 across the five test folds.

### 3.3. Qualitative evaluation of attention coefficients

**Figure 2.**
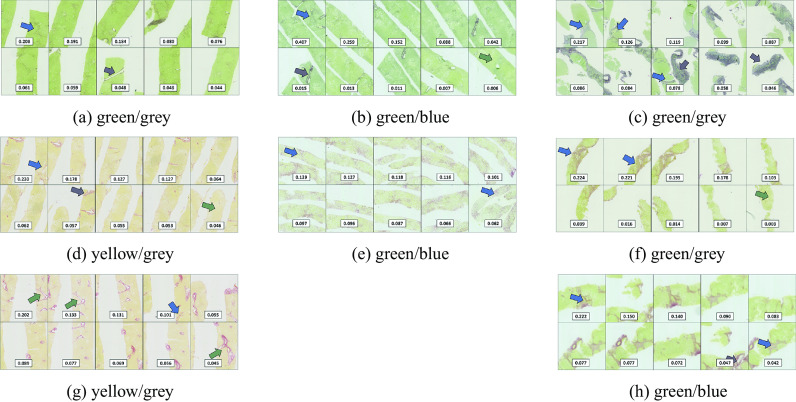
True positive (TP) cases of severe fibrosis illustrated on tiles with the 10 highest attention weights. WSI color categories are indicated under each case. Arrows color-coding: blue = relevant pathological signs of bridging, purple = red artefacts, green = healthy portal tracts. (a–f) Cases with high attention correctly focused on fibrotic signs as well as on artifacts and portal tracts. (g) Case showing higher attention put on portal tracts sliced longitudinally than on fibrotic signs. (h) Case with strong blurring still showing high attention on fibrotic signs.

**Figure 3.**
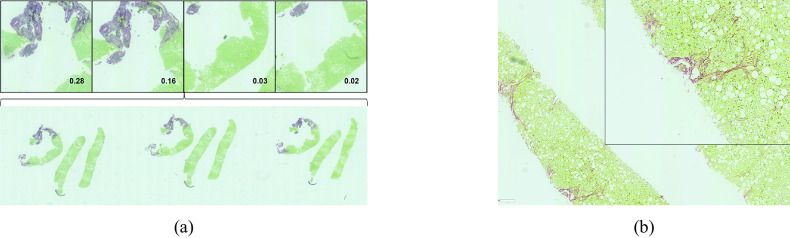
False positive (FP) cases of severe fibrosis. (a) Red artifacts: The tiles with two highest and two lowest attention weights show a focus on red pixels from a vein cut within the tissue. (b) Borderline case: This case was reconsidered to be a potential severe case by the expert clinician.

**Figure 4.**
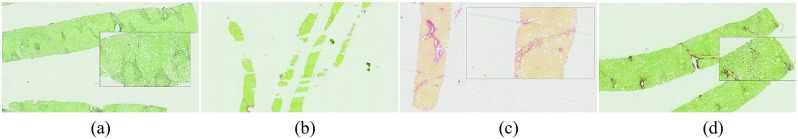
False Negative (FN) cases. (a) Faded red stain; (b) Poor-quality biopsy with tissue crumbling; (c,d) Borderline cases with sparse bridging signs shown in zoomed boxes: (c) most red pixels correspond to healthy portal tracts sliced longitudinally; (d) Very thin bridging patterns.

Our best model (SE-ResNet18 + Gated Attention + Dataloader RAND-n train/test) outputs attention weights for all tiles inside bags, which can be interpreted as the importance given by the network to a given tile to make the final inference at the bag level. We reviewed tiles with high and low attention weights with three clinical experts. For positive cases, we focused separately on true-positive (TP–Figure 2) and false-positive (FP–Figure 3) cases. We also reviewed whole WSIs for false-negative (FN–Figure 4) cases to analyze the missed pathological signs.

This extensive review leads to the following:

General observations:

1. Tiles with high attention scores in TP cases are usually of high diagnostic significance with the confirmed presence of fibrotic patterns (cf. Figure 2).
2. The presence of large red artifacts in TP cases, such as vein tissue in cirrhotic cases biopsied via wires (Figure 2c) did not distract the model to pay high attention to fibrotic patterns.
3. Some common characteristics seem to explain the majority of cases classified incorrectly by our model, (cf. Figures 3 and 4), detailed in the next paragraph.

Going now into more details on errors patterns:

1. False-positive WSIs generally fall into two categories: (a) misleading large red artifacts such as a vein cut lengthwise (Figure 3a) shown in all tiles with high attention (i.e., focus on a single large region in the WSI); (b) borderline cases reclassified as a severe case after reviewing the highest attention weighted tiles (Figure 3b).
2. False-negative WSIs have four key characteristics: (a) faded stain and loss of color contrast on old WSIs in color categories green/grey and green/blue (Figure 4a) which would require higher magnification detect fibrosis; (b) poor-quality biopsy due to tissue crumbling in advanced fibrosis cases (Figure 4b); (c) cases with very delicate fibrosis or very sparse pathological signs (Figure 4c,d). In such cases, high attention was correctly assigned to tiles with healthy tissue. Case in Figure 4c corresponds to a WSI that is overall healthy except for a single, borderline bridge in the zoomed box. It is interesting to note on this case that red structures from the lumen of blood vessels cut longitudinally at branching points were not misinterpreted as pathological signs. Case in Figure 4d corresponds to a WSI where fibrosis bridging is only a few cells thick; There was also a case where a large piece of portal tract was surrounded by faint fibrosis; (d) WSIs in color category yellow/grey are more often misclassified as healthy tissue, likely due to the small sample size.

## 4. Conclusions

In this study, we have proposed an end-to-end weakly-supervised multiple-instance DL framework that can distinguish severe from mild fibrosis on histopathological WSIs from a retrospective clinical dataset stained with Sirius Red. To the best of our knowledge, this is the first study applying DL on Sirius-Red stained WSIs to stage fibrosis.

We designed our method to handle multiple challenges in this new application: small image cohort, sparse signs on large tissue samples, large stain variability and presence of visual artifacts, a priori knowledge on the red color of pathological signs of interest (e.g., bridging).

Our key contributions are as follows: We first demonstrated the benefit of using two advanced concepts (squeeze-excitation encoder and gated attention pooling) when training on one bag per WSI composed of tiles with high red-pixel content. These first experiments lead to an average baseline Accuracy of 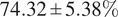 and an average F1 score of 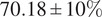 .

We further demonstrated the benefit of switching to *multiple inferences* on multiple bags per WSI for our problem. Our final model has an average accuracy of 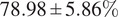 , and an average F1 score of 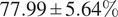 and an AUC of 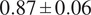 on the LiFib dataset. Finally, we showed some clinical interest in reviewing tiles with high attention weights, which lead to one case reclassified as severe.

Our results compare well with the performance reported on fibrosis staging using a different stain and more annotations^(^^32^ ^)^. Our results are also on par with an NAFLD scoring solution trained with WSI-level annotations on H&E and Trichrome stained biopsies^(^^21^ ^)^.

We acknowledge three limitations in our work. First, we relied on a single expert for ground truth annotations, while visual fibrosis stage scoring on WSIs has high inter- and intra- clinical expert variability. Second, GPU constraints limited batch and bag size ranges that could be tested. Finally, we conducted minimal pre-processing on our clinical dataset.

Based on an extensive visual review of our results with three expert clinicians, we have identified crucial points to be addressed in future work such as (a) training with multiple zoom factors, (b) forcing diversity of localization of tiles in each bag to avoid domination effect of large red artifacts, (c) pre-identifying cases with crumbling tissue and adapting the training procedure to give more importance to such challenging cases. Future investigations could also incorporate additional preprocessing steps^(^^31^ ^)^, stain normalization on Sirius-Red stained images^(^^27^ ^)^, and extend the model to multi-class classification. We could also consider multimodal learning, since reasonable NAFLD diagnosis performance has been reported using DL on non-imaging data^(^^36^ ^,^^37^ ^)^.

Replication code and pretrained models are available at https://github.com/s-n-naik/Deep-Learning-for-Sirius-Red-Stained-Histopatholological-Images.

## Data Availability

The replication code is available at https://github.com/s-n-naik/Deep-Learning-for-Sirius-Red-Stained-Histopatholological-Images.
